# Rates of Post-Partum Psychosis in women with risk factors cared for by a specialist community perinatal mental health service in London

**DOI:** 10.1192/j.eurpsy.2022.2228

**Published:** 2022-09-01

**Authors:** T. Tsoumpris, K. Tang, M. Miele, C. Acosta

**Affiliations:** 1 Central and North West London NHS Foundation Trust, Brent And Harrow Community Perinatal Mental Health Service, London, United Kingdom; 2 St Mary’s Hospital, Psychiatry, London, United Kingdom; 3 Central and North West London NHS Foundation Trust, Perinatal Mental Health Service, Chelsea & Westminster Hospital, London, United Kingdom

**Keywords:** Post-Partum Psychosis, Mother and Baby Unit, Bipolar Affective Disorder, Perinatal

## Abstract

**Introduction:**

Community Perinatal Mental Health Services (CPMHS) have been established in the UK, however, there is limited research around their real-world effectiveness. Post-Partum Psychosis (PPP), a severe episode of affective psychosis usually occurring soon after birth, has known risk factors. CPMHS offer assessment and interventions for women with risk factors for PPP, with a view to reducing the risk of its occurrence, as well as, where necessary, to proactively manage the illness to minimise the impact on the mother-infant dyad, as well as associated risks to self and/or others.

**Objectives:**

To review the rate of PPP in women with established risk factors, who were referred and managed by our CPMHS between September 2019-September 2021. This rate will be compared with the known rates of PPP reported in the literature. Rates of non-psychotic relapse, acute hospitalisation, children social care supervision and mother-infant separation as a result of postnatal relapse will be (amongst others) secondary outcomes. Perinatal interventions offered to reduce the risk of PPP and contingency planning will also be reviewed.

**Methods:**

This will be a retrospective case review study involving women referred and cared for by our CPMHS from October 2019 to October 2021, with known risk factors for PPP. Women identified as high risk for PPP receive consultant led-care in our service, therefore cases will be identified via the individual caseloads. Subsequently, electronic case notes will be reviewed to determine the primary and secondary outcomes, as well as the perinatal interventions that were offered.

**Results:**

To be reported.

**Conclusions:**

To be reported.

**Disclosure:**

No significant relationships.

